# In Vitro Assays to Identify Metabolism-Disrupting Chemicals with Diabetogenic Activity in a Human Pancreatic β-Cell Model

**DOI:** 10.3390/ijms23095040

**Published:** 2022-05-01

**Authors:** Reinaldo Sousa Dos Santos, Regla María Medina-Gali, Ignacio Babiloni-Chust, Laura Marroqui, Angel Nadal

**Affiliations:** 1 Instituto de Investigación, Desarrollo e Innovación en Biotecnología Sanitaria de Elche (IDiBE), Universidad Miguel Hernández de Elche, 03202 Elche, Spain; r.sousa@umh.es (R.S.D.S.); rmedina@umh.es (R.M.M.-G.); ibabiloni@umh.es (I.B.-C.); lmarroqui@umh.es (L.M.); 2 CIBER de Diabetes y Enfermedades Metabólicas Asociadas (CIBERDEM), Instituto de Salud Carlos III, 28029 Madrid, Spain

**Keywords:** apoptosis, diabetes, endocrine disruptors, insulin secretion, metabolism disrupting chemicals, pancreatic β-cells, test methods

## Abstract

There is a need to develop identification tests for Metabolism Disrupting Chemicals (MDCs) with diabetogenic activity. Here we used the human EndoC-βH1 β-cell line, the rat β-cell line INS-1E and dispersed mouse islet cells to assess the effects of endocrine disruptors on cell viability and glucose-stimulated insulin secretion (GSIS). We tested six chemicals at concentrations within human exposure (from 0.1 pM to 1 µM). Bisphenol-A (BPA) and tributyltin (TBT) were used as controls while four other chemicals, namely perfluorooctanoic acid (PFOA), triphenylphosphate (TPP), triclosan (TCS) and dichlorodiphenyldichloroethylene (DDE), were used as “unknowns”. Regarding cell viability, BPA and TBT increased cell death as previously observed. Their mode of action involved the activation of estrogen receptors and PPARγ, respectively. ROS production was a consistent key event in BPA-and TBT-treated cells. None of the other MDCs tested modified viability or ROS production. Concerning GSIS, TBT increased insulin secretion while BPA produced no effects. PFOA decreased GSIS, suggesting that this chemical could be a “new” diabetogenic agent. Our results indicate that the EndoC-βH1 cell line is a suitable human β-cell model for testing diabetogenic MDCs. Optimization of the test methods proposed here could be incorporated into a set of protocols for the identification of MDCs.

## 1. Introduction

Diabetes prevalence has been continuously growing in recent decades, reaching pandemic proportions [1]. Genetic and environmental factors play a role in diabetes etiology. While the genetic background may predispose individuals to the disease, environmental factors, including exposure to chemical pollutants that interfere with the endocrine system (also known as endocrine-disrupting chemicals or EDCs), may act as triggers to diabetes development. Type 1 diabetes is an autoimmune disease characterized by pancreatic β-cell apoptosis as the result of an immune attack [2,3]. Although it is still unclear whether there is a link between type 1 diabetes and exposure to EDCs, some evidence indicates that EDC exposure, especially during development, may play a role in the pathogenesis of type 1 diabetes [4]. Type 2 diabetes is the result of β-cell dysfunction and eventually death, which usually happen in a background of insulin resistance. β-cell dysfunction represents an early phenomenon in the development of type 2 diabetes as it occurs before dysglycemia advances [5]. In some special cases, insulin hypersecretion may also lead to dysglycemia [5,6]. After two decades of research, the relationship between exposure to EDCs and type 2 diabetes development has grown sufficiently strong to consider these environmental pollutants as a potential cause of type 2 diabetes [7,8,9].

Metabolism disrupting chemicals (MDCs) are defined as endocrine disruptors that alter susceptibility to metabolic disorders, including diabetes, obesity and non-alcoholic fatty liver disease [8,10]. Despite growing evidence suggesting a relationship between exposure to MDCs and susceptibility to metabolic diseases in cellular and animal models as well an in epidemiological studies, there is a lack of test methods to identify potential MDCs [11]. GOLIATH is a project within the Horizon 2020 programme of the European Commission whose aim is to address the urgent need to develop test protocols that allow the identification of MDCs that cause relevant toxic effects through an endocrine mode of action [11]. One of our objectives within the GOLIATH project is to design test methods to identify MDCs that may pose a risk for the development of diabetes.

As the insulin produced and secreted by β-cells is the only hormone in our body that lowers blood glucose levels, any disruption in its secretion (e.g., due to β-cell malfunction and/or death) poses a serious risk for diabetes onset. Therefore, it is essential to develop robust tests to detect MDCs that might decrease β-cell viability and/or disrupt its function. In the present work we sought to investigate in vitro test methods that could allow the identification of metabolism disrupting activity in β-cells. As test methods, MTT assay and staining with DNA-binding dyes were employed to evaluate β-cell viability, while glucose-stimulated insulin secretion (GSIS) and mRNA expression by real time PCR were used to assess β-cell function. We used two β-cell lines, namely the rat insulinoma-derived INS-1E [12] and the human EndoC-βH1 [13]. INS-1E cells are a stable β-cell line surrogate with less heterogeneity than the non-clonal INS-1 cell line from which INS-1E were cloned [12]. EndoC-βH1 cells present similar metabolic function to INS-1 832/13 and similar insulin secretion in response to glucose in comparison to human islets; this means that EndoC-βH1 cells are as useful as INS-1 cells but with the advantage of being from human origin [14]. Both cell lines were exposed to six MDCs, namely Bisphenol A (BPA), Tributyltin (TBT), Perfluorooctanoic acid (PFOA), Triphenylphosphate (TPP), Triclosan (TCS) and Dichlorodiphenyldichloroethylene (DDE). These chemicals were selected because they have a wide range of mechanisms of action, and there are existing data from human and/or animal studies that support metabolism disrupting effects following exposure [11]. The concentration range tested herein was based on biomonitoring studies for BPA [15], TBT [16], PFOA [17], TPP [18], TCS [19] and DDE [20]. For more details regarding these compounds (e.g., chemical structures and evidence of their actions as MDCs), please refer to Ref. [11].

BPA and TBT have been previously tested in β-cells. BPA and TBT decreased β-cell viability in different β-cell models, namely INS-1E cells [21] and RINm5F cells [22]. Moreover, both chemicals have been characterized as MDCs that alter insulin secretion in primary mouse islets/β-cells [22,23,24], rat islets and insulinomas [25,26,27] as well as human islets [28,29]. Therefore, we used these two chemicals as positive controls to evaluate our human β-cell model. Despite some evidence of their potential as MDCs, the other four chemicals, PFOA, TPP, TCS and DDE, were considered “unknown” chemicals due to the lack of data about their effects on β-cells. To test these chemicals, we followed an adverse outcome pathway framework, in which we studied the molecular initiated event (MIE) by pharmacology and two key events, namely gene expression and reactive oxygen species (ROS) production. Finally, we evaluated β-cell viability and GSIS as adverse effects. 

## 2. Results

### 2.1. BPA and TBT Induce β-Cell Death

**Figure 1:**
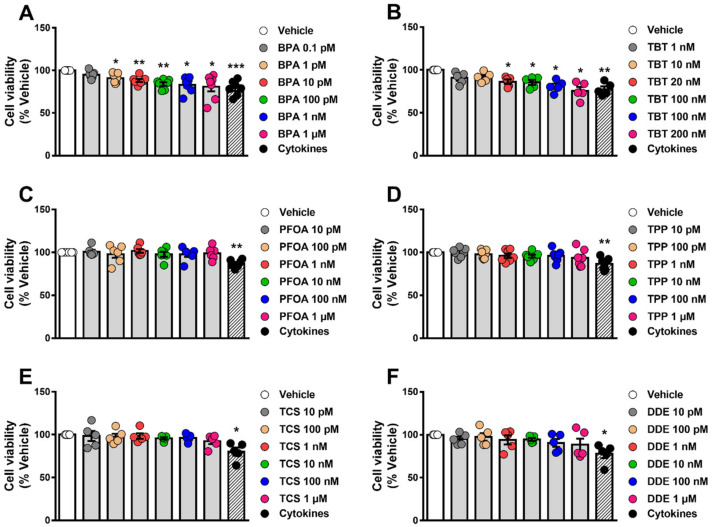
β-cell viability upon MDC exposure. EndoC-βH1 cells were treated with vehicle (DMSO) or different doses of BPA (**A**), TBT (**B**), PFOA (**C**), TPP (**D**), TCS (**E**) or DDE (**F**) for 48 h. A cocktail of the cytokines IL-1β + IFNγ (50 and 1000 U/mL, respectively) was used as a positive control. Cell viability was evaluated by MTT assay. Results are expressed as % vehicle-treated cells. Data are shown as means ± SEM (n = 5–7 independent experiments, where each dot represents an independent experiment). * p ≤ 0.05, ** p ≤ 0.01 and *** p ≤ 0.001 vs. Vehicle. MDCs vs. Vehicle by one-way ANOVA; Cytokines vs. Vehicle by two-tailed Student’s *t* test.

To assess whether different MDCs could affect cell survival, we measured cell viability by two different methods, namely the MTT assay and staining with the DNA-binding dyes Hoechst 33342 and propidium iodide (HO/PI). The former is a colorimetric method that indicates cell viability based on metabolic activity [30,31], while the latter is a fluorescent method that allows discrimination between viable, apoptotic and necrotic cells [32]. We exposed EndoC-βH1 and INS-1E cells to a range of concentrations of each MDC and cell viability was measured by MTT assay 48 h and 72 h following treatment (Figure 1, Supplementary Materials Figure S1). Of the six chemicals tested, only BPA (Figure 1A, Supplementary Materials Figure S1A) and TBT (Figure 1B, Supplementary Materials Figure S1B) decreased cell viability, both acting in a dose-dependent manner. When compared to their respective vehicles, the highest BPA dose (i.e., 1 μM) induced a 15–20% decrease in viability, whereas the highest TBT dose (i.e., 200 nM) decreased viability by 24–27% after exposure for 48 h, depending on the cell model used.

**Figure 2:**
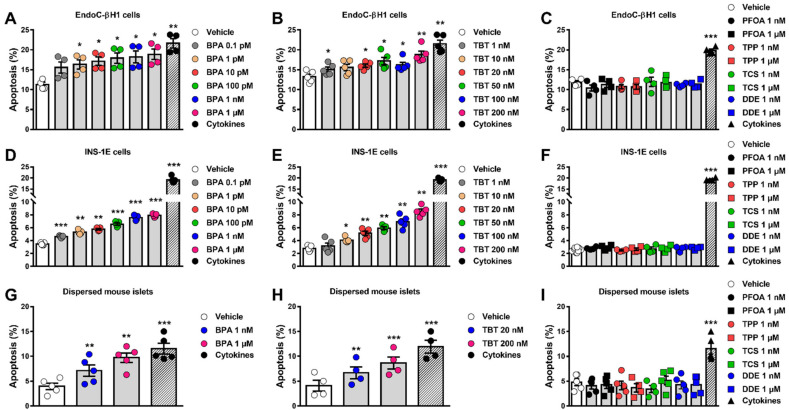
β-cell apoptosis upon MDC exposure. EndoC-βH1 (**A**–**C**) and INS-1E cells (**D**–**F**) or dispersed mouse islets (**G**–**I**) were treated with vehicle (DMSO) or different doses of BPA (**A**,**D**,**G**), TBT (**B**,**E**,**H**), PFOA, TPP, TCS or DDE (**C**,**F**, **I**) for 24 h (**A**–**F**) or 48 h (**G**–**I**). A cocktail of the cytokines IL-1β + IFNγ (10 and 100 U/mL, respectively for INS-1E cells; 50 and 1000 U/mL, respectively, for EndoC-βH1 cells and dispersed mouse islets) was used as a positive control. Apoptosis was evaluated using HO and PI staining. Data are shown as means ± SEM (n = 4–5 independent experiments, where each dot represents an independent experiment). * p ≤ 0.05, ** p ≤ 0.01 and *** p ≤ 0.001 vs. Vehicle. MDCs vs. Vehicle by one-way ANOVA; Cytokines vs. Vehicle by two-tailed Student’s *t* test.

As seen by the MTT assay, assessment of β-cell viability by HO/PI showed that BPA and TBT induced apoptosis in EndoC-βH1 and INS-1E cells upon 24 h treatment, whereas doses up to 1 µM of PFOA, TPP, TCS and DDE did not affect the viability of these cells (Figure 2, Supplementary Materials Figure S2A–D). Interestingly, higher concentrations of PFOA (20 to 200 µM) induced apoptosis in EndoC-βH1 and INS-1E cells (Supplementary Materials Figure S2E,F). Due to the sensitivity of this method, we observed that BPA doses within the picomolar range increased apoptosis in both cell lines (Figure 2A,D). To compare these results with a more physiological cell system, we used dispersed mouse islet cells. As observed in both β-cell lines, BPA and TBT also induced apoptosis in dispersed islets (Figure 2G,H), while PFOA, TPP, TCS and DDE did not induce apoptosis at the doses tested (Figure 2I). Depending on the cell model, 1 μM BPA induced 1.7 to 2.5-fold increase in apoptosis, while 200 nM TBT promoted a 1.5- to 3-fold increase in apoptosis. These findings indicate that MDCs affect the viability of the human EndoC-βH1 cells, the rat line INS-1E and the primary mouse β-cells in a very similar way. Based on the findings described above, we used only EndoC-βH1 cells to confirm our results with a third method, namely caspase 3/7 activity assay. We observed that caspase 3/7 activity was augmented by BPA and TBT exposure but no by exposure to PFOA, TPP, TCS and DDE (Supplementary Materials Figure S3).

A cocktail of proinflammatory cytokines, namely interleukin-1β (IL-1β) and interferon-γ (IFNγ), was used as a positive control. As expected, this mix of cytokines decreased viability, induced apoptosis or increased caspase 3/7 activity in all models tested (Figure 1 and Figure 2, Supplementary Materials Figures S1 and S3).

### 2.2. Estrogen Receptors Are Involved in BPA-Induced β-Cell Apoptosis

**Figure 3:**
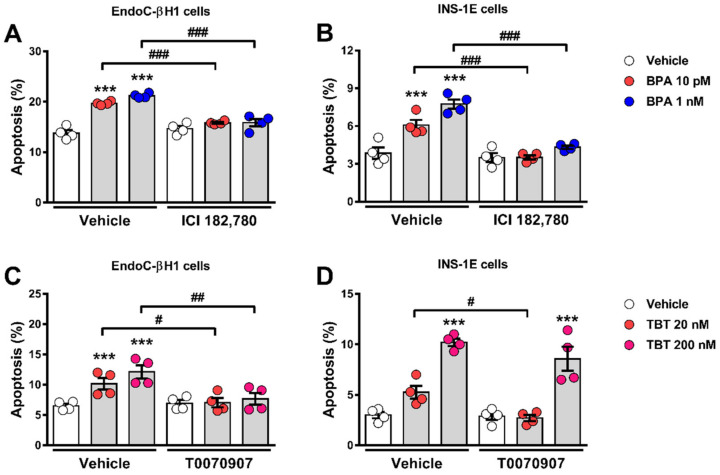
Estrogen receptors and PPARγ are involved in BPA-and TBT-induced β-cell apoptosis, respectively. EndoC-βH1 (**A**) and INS-1E (**B**) cells were treated with vehicle (DMSO) or BPA (10 pM or 1 nM) in the absence or presence of 1 µM ICI 182,780 for 24 h. EndoC-βH1 (**C**) and INS-1E (**D**) cells were treated with vehicle (DMSO) or TBT (20 nM or 200 nM) in the absence or presence of 100 nM T0070907 for 24 h. Apoptosis was evaluated using HO and PI staining. Data are shown as means ± SEM (n = 4 independent experiments, where each dot represents an independent experiment). *** p ≤ 0.001 vs. its respective Vehicle; *^#^ p* ≤ 0.05, *^##^ p* ≤ 0.01 and *^###^ p* ≤ 0.001 as indicated by bars. Two-way ANOVA.

In β-cells, BPA effects on insulin content and secretion as well as in apoptosis involve the activation of estrogen receptors ERα and ERβ [23,28,33]. Figure 3A,B show that the pure estrogen receptors antagonist ICI 182,780 completely blocked BPA-induced apoptosis in EndoC-βH1 (Figure 3A) and INS-1E cells (Figure 3B). These results indicate that the MIE responsible for the BPA-induced β-cell death described in Figure 1 and Figure 2 involves estrogen receptors.

### 2.3. PPARγ Is Involved in TBT-Induced β-cell Apoptosis

TBT acts as an agonist of both the peroxisome proliferator-activated receptor γ (PPARγ) and retinoid X receptors (RXRs) [34,35]. To assess whether PPARγ activation was part of the MIE whereby TBT induces apoptosis, we used the PPARγ antagonist T0070907. In EndoC-βH1 cells, T0070907 blocked TBT-induced apoptosis at both doses (Figure 3C), while in INS-1E cells, T0070907 prevented the apoptotic effect of TBT at a low (20 nM) but not at a high dose (200 nM) (Figure 3D).

Rosiglitazone, a well-known PPARγ-selective activator, not only did not induce apoptosis, but it also abrogated TBT-induced apoptosis in EndoC-βH1 and INS-1E cells (Supplementary Materials Figure S4). These findings suggest that PPARγ activation is part of the MIE whereby TBT induces β-cell apoptosis and show that two PPARγ agonists, rosiglitazone and TBT, have opposite effects on β-cell viability.

### 2.4. BPA and TBT, but Not PFOA, Induce ROS Generation

**Figure 4:**
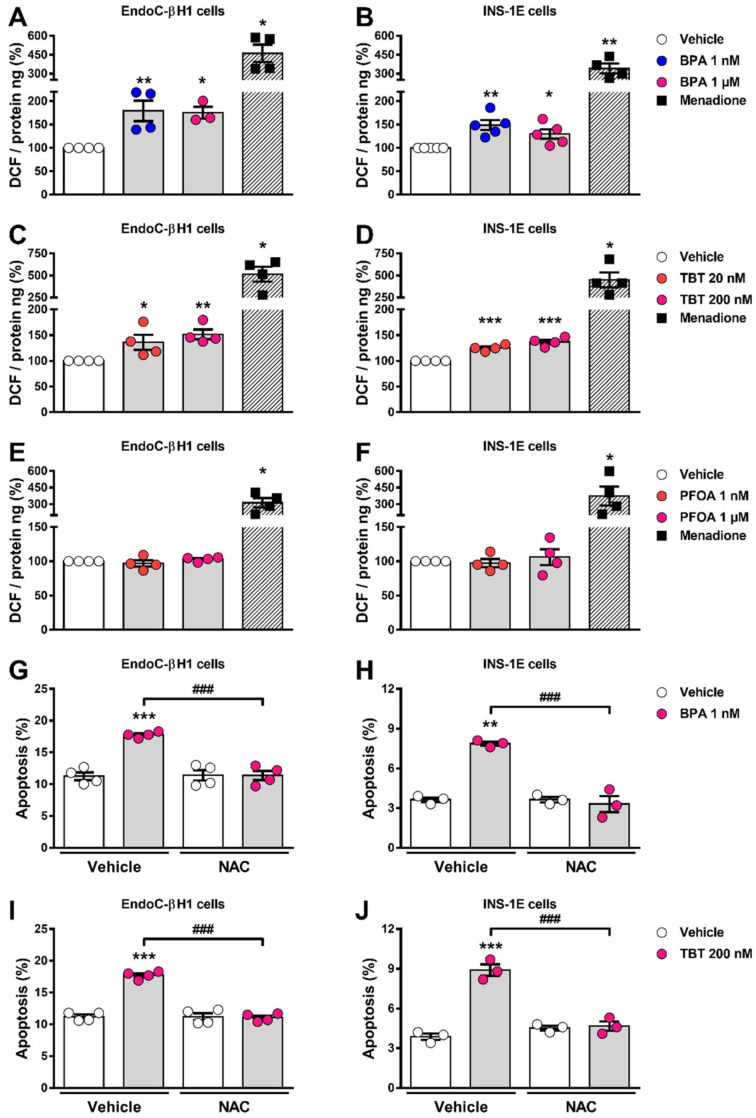
ROS production upon MDC exposure. EndoC-βH1 (**A**,**C**,**E**), and INS-1E (**B**,**D**,**F**) cells were treated with vehicle (DMSO) or different doses of BPA (**A**,**B**), TBT (**C**,**D**) or PFOA (**E**,**F**) for 24 h. Menadione (15 μM for 90 min) was used as a positive control. ROS production was measured by oxidation of the fluorescent probe 2′,7′-dichlorofluorescein diacetate (DCF) and normalized by total protein. Results are expressed as % vehicle-treated cells. EndoC-βH1 (**G**,**I**) and INS-1E (**H**,**J**) cells were treated with vehicle (DMSO), BPA (1 μM; **G**,**H**) or TBT (200 nM; **I**,**J**) in the absence or presence of N-acetylcysteine (NAC; 3 mM) for 24 h. Apoptosis was evaluated using HO and PI staining. Data are shown as means ± SEM (n = 3–5 independent experiments, where each dot represents an independent experiment). (**A**–**F**) * p ≤ 0.05, ** p ≤ 0.01 and *** p ≤ 0.001 vs. Vehicle. MDCs vs. Vehicle by one-way ANOVA; Menadione vs. Vehicle by two-tailed Student’s *t* test. (**G**–**J**) ** p ≤ 0.01 and *** p ≤ 0.001 vs. its respective Vehicle; *^###^ p* ≤ 0.001 as indicated by bars. Two-way ANOVA.

Previous work demonstrated that both BPA and TBT induced oxidative stress in β-cells [21,25,33]. In line with these findings, we observed that BPA and TBT induced ROS generation in both cell lines explored here (Figure 4A–D). PFOA, which did not induce apoptosis at any of the concentrations used, failed to produce ROS at both low (1 nM) and high (1 µM) doses (Figure 4E,F). Menadione, which is known for inducing ROS production, was used as a positive control. Treatment with the ROS scavenger N-acetylcysteine prevented BPA- and TBT-induced apoptosis in EndoC-βH1 and INS-1E cells (Figure 4G–J), which indicates that ROS production is a key event involved in BPA- and TBT-induced β-cell apoptosis.

### 2.5. β-Cell Function Is Disturbed by Different MDCs

**Figure 5:**
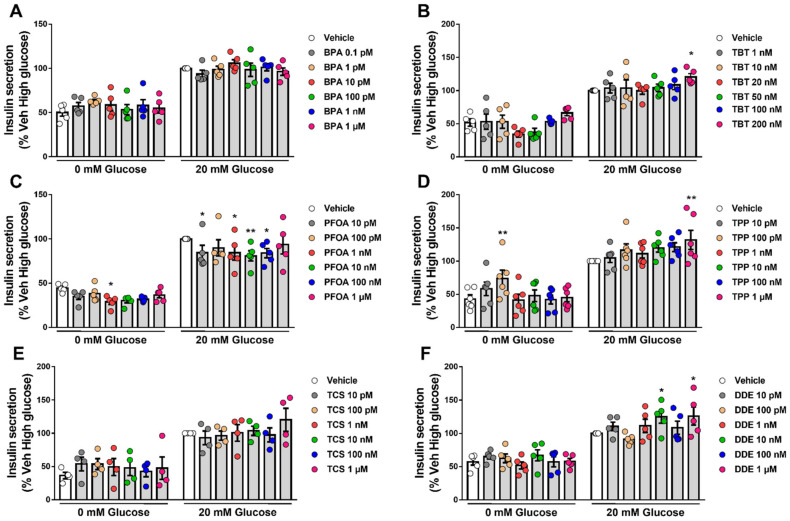
Glucose-stimulated insulin secretion upon MDC exposure. EndoC-βH1 cells were treated with vehicle (DMSO) or different doses of BPA (**A**), TBT (**B**), PFOA (**C**), TPP (**D**), TCS (**E**) or DDE (**F**) for 48 h. Insulin secretion was measured at 0 and 20 mM glucose, and insulin released into the medium was measured by ELISA. Data are normalized to insulin secretion at high glucose (20 mM) in vehicle-treated cells (considered as 100%). Data are shown as means ± SEM (n = 4–6 independent experiments, where each dot represents an independent experiment). * p ≤ 0.05 and ** *p* ≤ 0.01 vs. its respective Vehicle. Two-way ANOVA.

Our next step was to explore whether the six MDCs tested herein could perturb β-cell function. For this purpose, we measured GSIS and insulin content in EndoC-βH1 cells upon exposure to different doses of each MDC for 48 h (Figure 5). BPA (Figure 5A) and TCS (Figure 5E) did not modify GSIS, despite a trend observed in cells treated with 1 μM TCS. An increase in insulin secretion was observed upon exposure to the highest doses of TBT (200 nM; Figure 5B), TPP (1 μM; Figure 5D), and DDE (1 μM; Figure 5F). Curiously, we also observed an increase in insulin secretion at low glucose in EndoC-βH1 cells treated with 100 pM TPP (Figure 5D). PFOA promoted the most changes in β-cell function as exposure to several doses of this MDC decreased insulin secretion both at low and high glucose concentrations (Figure 5C). Interestingly, we found that PFOA modulated insulin secretion in a non-monotonic dose response-dependent manner, in which exposure from 10 pM to 100 nM PFOA reduced insulin secretion, while exposure to 1 µM did not significantly changed secretion (Figure 5C).

Exposure to BPA, PFOA, TPP, TCS and DDE did not modify insulin content (Supplementary Materials Figure S5); on the contrary, TBT promoted a slight increase at 20 nM and a 20% decrease in insulin content at 200 nM (Supplementary Materials Figure S5).

### 2.6. Effects of MDCs on β-Cell Gene Expression

**Figure 6:**
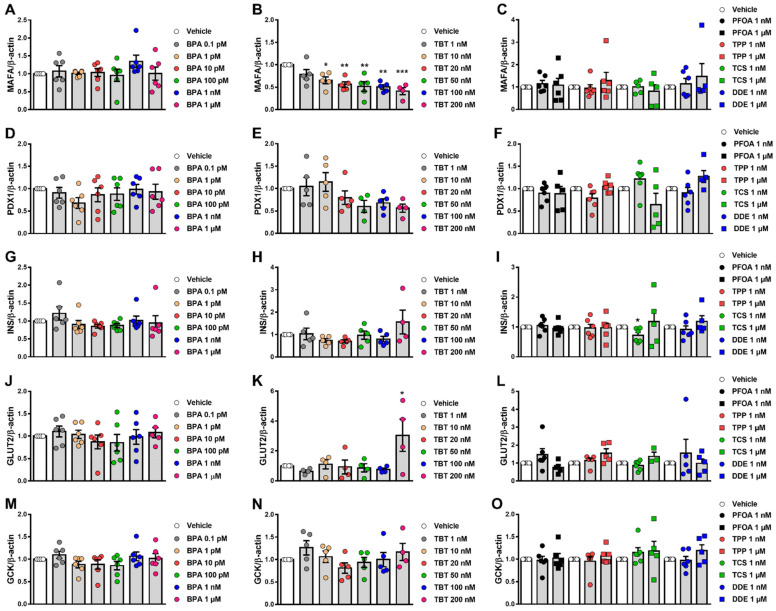
Gene expression upon MDC exposure in EndoC-βH1 cells. mRNA expression of MAFA (**A**–**C**), PDX1 (**D**–**F**), INS (**G**–**I**), GLUT2 (**J**–**L**) and GCK (**M**–**O**) was measured in EndoC-βH1 cells treated with vehicle (DMSO) or different doses of BPA (**A**,**D**,**G**,**J**,**M**), TBT (**B**,**E**,**H**,**K**,**N**), PFOA, TPP, TCS or DDE (**C**,**F**,**I**,**L**,**O**) for 24 h. mRNA expression was measured by qRT-PCR and normalized to the housekeeping gene β-actin, and then by vehicle-treated cells (considered as 1). Data are shown as means ± SEM (n = 4–6 independent experiments, where each dot represents an independent experiment). * p ≤ 0.05, ** p ≤ 0.01 and *** p ≤ 0.001 vs. its respective Vehicle. One-way ANOVA.

Finally, we examined whether the different MDCs could modulate the expression of some genes that are important for the appropriate β-cell phenotype and function, namely MAFA, PDX1, INS, GLUT2 and GCK. MAFA is a transcription factor that regulates the expression of genes implicated in insulin biosynthesis and secretion (e.g., PDX1 and GCK) [36,37,38,39]. PDX1 is a master transcription factor essential for pancreas development, β-cell maturation and identity as well as β-cell function by regulating the expression of several genes, including INS, GLUT2 and GCK [40,41,42,43,44]. Insulin, a peptide hormone synthesized and released by β-cells, is a critical regulator of metabolism and the only hormone in our body that lowers blood glucose levels [45]. GLUT2 is a glucose transporter required for glucose-stimulated insulin secretion [46], while GCK catalyzes glucose phosphorylation, the first rate-limiting step of glycolysis, and participates in glucose homeostasis by regulating the rate of insulin secretion [47]. After 24 h exposure, mRNA expression was measured in EndoC-βH1 and INS-1E cells. TBT significantly decreased MAFA expression, reaching about 50% inhibition in EndoC-βH1 and INS-1E cells (Figure 6B, Supplementary Materials Figure S6B). In EndoC-βH1 cells, TBT also induced a trend towards downregulation of PDX1, but these results did not reach statistical significance (Figure 6E). Of note, Pdx1 expression decreased upon exposure to 200 nM TBT in INS-1E cells (Supplementary Materials Figure S6E). In addition, TBT increased GLUT2 expression in EndoC-βH1 cells but not in INS-1E cells (Figure 6K, Supplementary Materials Figure S6N). No major significant changes in gene expression were observed when cell lines were treated with BPA, PFOA, TPP, TCS and DDE (Figure 6, Supplementary Materials Figure S6).

## 3. Discussion

Diabetes develops because there is a decrease in functional pancreatic β-cell mass, which may occur after disruption of β-cell secretory capacity, β-cell death or both. Therefore, it is plausible to say that GSIS and β-cell viability are the two main deterministic endpoints to study diabetogenic factors.

Our main objective in this work was to test and validate whether the EndoC-βH1 cell line would be an effective human β-cell model for developing test methods to assess β-cell viability and function. The development of these tests in a human β-cell model will help to identify contaminants with the potential to cause diabetes through an endocrine mode of action. The human EndoC-βH1 cell line has been used worldwide as a useful tool to increase our understanding of human islet biology and diabetes [48,49]. These cells recapitulate features of adult primary β-cells, and their open chromatin, transcriptomics and proteome landscapes were reported to be very similar to adult human β-cells [50,51]. In fact, EndoC-βH1 cells express all genes and proteins that lead to the phenotype of a typical human β-cell [52], including a similar set of ion channels, electrical activity and calcium signaling in response to glucose as compared with primary human β-cells [53]. Moreover, these cells are an appropriate human β-cell model for novel drug screening [54]. Nevertheless, despite representing a robust and reproducible cell system particularly useful for screening before advancing to studies in primary human β-cells, it is important to keep in mind that EndoC-βH1 cells present limitations as compared with primary cells [48]. For instance, the EndoC-βH1 cell line contains around 5–10% of the insulin content present in native human β-cells [13,54] and the expression of some β-cell markers and disallowed genes also differ between EndoC-βH1 cells and human islets [54]. Additionally, EndoC-βH1 cells show an elevated proliferation rate, whereas proliferation is an extremely rare event in adult human β-cells [51,55].

For the experimental protocols used in the present work we followed the recommendations of Univercell-Biosolutions, the company that supplies the EndoC-βH1 cells, including all culture media. This cell culture protocol has been previously used in key studies validating EndoC-βH1 cells as a suitable model of human β-cells [52,53,54,56,57].

As previous works have shown that BPA and TBT affect β-cell viability and function in rodent β-cell lines and mouse β-cells, we compared our results in EndoC-βH1 cells with those obtained in the rat INS-1E cell line and in dispersed cells isolated from mouse islets.

### 3.1. β-Cell Viability Tests

Our results showed that BPA and TBT induced β-cell death, whereas PFOA, TPP, TCS and DDE did not modify cell survival at the concentrations tested. In both cell lines studied, similar results were obtained using two different approaches to measure β-cell viability, namely the MTT assay and HO/PI DNA-binding dyes, and two time points for MTT assay (48 and 72 h). We confirmed our HO/PI findings in dispersed mouse islet cells. Moreover, we used a third method, caspase 3/7 activity assay, to confirm our data obtained in EndoC-βH1 cells. A cocktail of proinflammatory cytokines (IL-1β + IFNγ) normally employed in β-cell research [58,59] was used as a positive control to assure the methodology was working. Of note, EndoC-βH1 and INS-1E cells were exposed to different concentrations of cytokines because human and rat β-cells show different sensitivities to cytokines, where human β-cells are more resistant to cytokine-induced damage than rat β-cells [60,61].

There is plenty of evidence showing that BPA is an MDC that alter metabolism through an endocrine mode of action [7,62]. At nanomolar concentrations, BPA increased apoptosis following mitochondrial dysfunction in INS-1 cells [21,25]. Pancreatic β-cells from rodent pups perinatally treated with low doses of BPA (50 µg/kg/day, oral exposure) exhibited hypertrophy mitochondria and rough endoplasmic reticulum compared to control pups [63]. Male mouse pups prenatally treated with 10 µg/kg/day BPA showed lower pancreatic β-cell mass associated with increased caspase 3 activity and altered gene expression associated with mitochondrial function [64].

Our present findings regarding the effect of BPA on cell viability in EndoC-βH1, INS-1E and dispersed mouse islet cells mirrored previous data in β-cell models. In agreement with our previous data describing a crosstalk between the estrogen receptors G protein-coupled estrogen receptor 1, ERα and ERβ [33,65], BPA-induced apoptosis at low doses was abolished by the pure ERα and ERβ antagonist ICI 182,780. Thus, the MIE in the action of BPA involves estrogen receptors, including the G protein-coupled estrogen receptor 1, which predicts that chemicals that bind to these receptors should be further tested for adverse effects. Contrary to BPA, 17β-estradiol, the natural ligand of estrogen receptors, did not induce apoptosis; if anything, 17β-estradiol diminished it [33,65]. These data show that different estrogen receptor ligands may have opposite effects on β-cells, suggesting that ligand binding to estrogen receptors does not predict its effect on apoptosis, at least in β-cells. In line with previous in vivo [63,64] and in vitro [21,25,33,65] studies, we observed that BPA induced ROS production and that N-acetylcysteine abrogated BPA-induced apoptosis. These findings suggest that ROS generation might be considered an important key event that could predict β-cell death.

Our experiments indicate that TBT also induces apoptosis. In animal models, Zuo and colleagues showed that oral administration of 0.5, 5 and 50 μg/kg TBT every three days for 45 days induced apoptosis while inhibiting proliferation of mouse islet cells [66]. In rats, TBT effects on cell viability varied depending on the model studied. A TBT dose as low as 10 nM decreased cell viability by 20% in isolated rat islets [27], whereas doses up to 200 nM did not affect viability in the rat insulinoma cell line RINm5F [22,29]. Yet, exposure to 500 nM TBT for 24 h markedly increased the number of apoptotic cells in the RINm5F cell line [22]. In line with the results observed in mouse [66] and rat islets [27], here we show that doses from 1 to 200 nM reduced cell viability by inducing apoptosis in two cell lines, EndoC-βH1 and INS-1E, and in dispersed mouse islets. TBT is an agonist of PPARγ and RXR [34,35] and our results indicate that the apoptotic effect of TBT in β-cells is mediated by these receptors because it is abolished by a PPARγ antagonist. Like BPA, TBT also increased ROS levels, supporting a role for ROS production as a key event in the pathway to MDC-induced β-cell apoptosis. As previously reported in RINm5F cells [22], N-acetylcysteine prevented TBT-induced apoptosis in both cell lines tested herein. 

Besides BPA and TBT, exposure to the other four MDCs did not change β-cell viability at the doses tested. One of these EDCs, PFOA, did not change ROS production, confirming that assessment of ROS levels is a good predictor of apoptosis in our model. Regarding cell viability, our findings are in line with the studies available for PFOA and TCS. Following 48 h exposure, 1 µM PFOA did not induce apoptosis in rat RIN-m5F β-cells; only doses higher than 100 µM (up to 500 µM) decreased cell viability due to apoptosis [67]. Of note, we also found that higher concentrations of PFOA (20 to 200 µM) induced apoptosis in EndoC-βH1 and INS-1E cells. Regarding TCS, Ajao et al. found that 17 and 35 μM TCS induced β-cell death by necrosis in the mouse β-cell line MIN-6, whereas 7 μM TCS did not change MIN-6 viability [68]. This agrees with our results, in which 1 μM TCS did not change β-cell viability. Despite the lack of evidence in pancreatic β-cells, it has been shown that exposure to high doses of TPP and DDE (>10 μM) induced apoptosis in other cells and tissues: TPP in hepatocytes, epithelial tumoral JEG-3 cells from placenta and murine BV-2 microglia [69,70,71]; and DDE in Sertoli cells [72,73]. Exposure to high concentrations of *p,p′*-DDT (>150 μM) for 24 h decreased viability in NES2Y human pancreatic β-cell line [74]. Lack of publications with these EDCs at low concentrations does not allow comparison with the present findings. 

Finally, our present results indicate that EndoC-βH1 cells are a robust human β-cell model for identifying MDCs that alter β-cell viability. Furthermore, the methods used herein to measure cell viability and/or apoptosis are easy to use, particularly the MTT assay, and appear to be suitable for testing cell death in response to MDCs. Assessment of ROS production as a key event correctly predicted β-cell death, suggesting that it could be a useful surrogate for measurement of cell viability.

### 3.2. β-Cell Function Tests

INS-1E have been used for studies of β-cell secretion for the last two decades. They present a similar GSIS to rat islets with a maximal increase of about 6-fold at 15 mM glucose and a 50% effective concentration about 10 mM [12]. Its electrical activity is, however, different to that described for islet cells and it is saturated at a concentration of 7.5 mM glucose [12]. EndoC-βH1 is an appropriate model to study GSIS; these cells release more insulin at basal and stimulatory (20 mM) glucose levels than INS-1 832/13 cells and their stimulation index is comparable to human islets, i.e., a 2- to 2.5-fold increase in insulin secretion upon glucose stimulation [14]. We also report a 2- to 3-fold increase upon stimulation with 20 mM glucose, which is similar to what has been published in the literature [13,14,53,54], indicating that our cells respond adequately to glucose. Conversely, due to technical issues, we were unable to use INS-1E cells for analyses of GSIS and insulin content. Of note, an earlier study demonstrated that the rat β-cell line INS-1 832/13 seemed to lack certain characteristics that made it unsuitable as a screening system for diabetogenic contaminants [26].

Our results indicate that PFOA was the MDC that produced the most changes in insulin secretion in EndoC-βH1 cells at basal and glucose-stimulating levels. This MDC induced a decrease in GSIS at very low doses, within the pM and nM range while no effect was observed at 1 µM. Interestingly, neither apoptosis nor ROS production were induced upon exposure to 1 nM and 1 µM PFOA. These data suggest that the decrease in insulin release shown here is not due to a cytotoxic action of PFOA. We did not test higher PFOA concentrations because they are not within the range of human exposure. Nonetheless, a previous study demonstrated that higher concentrations of PFOA, above 10 µM, increased insulin release in the mouse-derived β-TC-6 β-cell line through the G-protein coupled receptor GPR40 [75]. Studies in animal models indicate that PFOA alters glucose homeostasis. In adult male mice, oral administration of PFOA 1.25 mg/kg for 28 days increased fasting blood glucose levels and decreased hepatic glycogen and glucose content, but did not influence insulin blood levels [76]. Higher PFOA doses (5 mg/kg) induced hyperinsulinemia in the fasted state [77]. In CD-1 mice offspring exposed in utero, low concentrations of PFOA (0.01–0.1mg/kg) increased weight gain as well as insulin and leptin levels in blood [78]. These alterations in insulin blood levels could be due to an indirect rather than a direct effect on β-cells. Unfortunately, we could not find any evidence of direct effects of low doses of PFOA on insulin secretion so that we could compare with our data. Although our results need further confirmation, they point to PFOA as a potential diabetogenic MDC. 

As a key event with the potential to predict alterations in β-cell function, we measured expression of five key genes involved in β-cell identity (MAFA and PDX1) and function (INS, GLUT2 and GCK). None of those genes changed with PFOA treatment, indicating that these genes may not be good candidates to predict alterations in β-cell function.

The other MDCs that changed GSIS were TBT and TPP at the highest dose tested and DDE at 10 nM and 1 µM. Previous work suggested that TBT at 100 and 200 nM increased GSIS in RIN-m5F cells and in human islets [29], which is in the same concentration range shown in our work. Chen et al., suggested that changes in intracellular Ca^2+^ concentrations, ROS levels and activation of the protein kinase C signaling pathway could be involved in TBT-induced insulin secretion [29]. Here we observed that an increase in GSIS promoted by 200 nM TBT correlated with an increase in the expression of the glucose transporter GLUT2. Notably, TBT decreased MAFA expression even at low concentrations in EndoC-βH1 and at 200 nM in INS-1E. MAFA controls glucose-responsive transcription of insulin and other genes in β-cells [44]. Insulin content decreased with 200 nM TBT, which might be, in part, due to MAFA downregulation. Insulin content, however, was not diminished upon treatment with TBT doses that decreased MAFA expression. On the contrary, 20 nM TBT increased insulin content. Then, even though our findings regarding MAFA expression are of interest, further research is required to link its downregulation to changes in insulin content. As gene expression and GSIS were measured at different time points (24 and 48 h, respectively), a causal relation between these two events cannot be firmly established. It is possible, however, that TBT increases insulin secretion by mediating changes in other proteins involved in GSIS, as we have previously reported for BPA [23,24,28,79,80].

Little is known about TPP effects on insulin and glucose homeostasis. In other metabolism-related cell systems, such as differentiated 3T3-L1 adipocytes, TPP exposure increased glucose uptake and lipolysis [81]. In animal models, perinatal exposure to TPP accelerated type 2 diabetes onset [82] and a mix of organophosphate flame retardants, including TPP, altered glucose homeostasis in an ERα-dependent manner [83]. Mice exposed to TPP during fetal development presented impaired glucose tolerance and increased insulin levels [84]. Here we show that exposure to 1 µM TPP induced an increase in insulin release, despite the lack of changes on gene expression at 1 nM or 1 µM. As this is the first time that a direct effect of TPP on β-cells is shown, we take our findings carefully until they can be further validated. 

DDE is a metabolite of the persistent endocrine disruptor DDT. Previous experiments performed in INS-1E cells showed that exposure to non-lethal concentrations of DDT or DDE changed insulin secretion and content/expression [74,85]. Upon 48 h treatment, low concentrations of DDT (1 fM to 1 µM) decreased GSIS in a non-monotonic manner, while all doses tested diminished intracellular insulin content [85]. In addition, INS-1E cells exposed for 1 month to a non-lethal concentration (10 µM) of DDT or DDE had lower mRNA levels of Ins1 and Ins2 as well as reduced expression of proinsulin and insulin [74]. On the other hand, DDE exposure increased insulin secretion in β-TC-6 cells [86], which agrees with our results that suggest an increase in GSIS at 10 nM and 1 µM DDE (insulin content remained unchanged in all concentrations tested). 

The findings described in the present work highly suggest that the EndoC-βH1 cell line is a valid human β-cell model to study the effect of MDCs on cell viability and function. We also suggest that the rat INS-1E cells may represent a valuable, alternative model for testing the effect of MDCs on cell viability because results observed in INS-1E cells mirror those obtained with EndoC-βH1 cells. Despite the similar findings observed between human and rat β-cell lines with respect to cell viability assays, it is important to keep in mind that human and rat β-cells may respond differently to the same stimulus. For instance, human β-cells are more resistant than rat β-cells to injury, including the deleterious effects of proinflammatory cytokines [60,61,87].

Altogether, we conclude that EndoC-βH1 may represent a valuable model of human β-cells for screening and testing procedures to identify MDC with diabetogenic activity. MTT assay and staining with DNA-binding dyes Hoechst 33342 and propidium iodide represent useful test methods to evaluate cell viability, while assessment of ROS production is a potential substitute for cell viability assays. Finally, measurement of GSIS seems a reliable test method to investigate MDC effects on β-cell function.

## 4. Materials and Methods

### 4.1. Chemicals

Chemicals used herein were purchased as follows: BPA (Cat. No. 239658), TBT (Cat. No. T50202), PFOA (Cat. No. 77262), TPP (Cat. No. 241288), TCS (Cat. No. PHR1338) and DDE (Cat. No. 123897) were obtained from Sigma-Aldrich (Barcelona, Spain). All stock solutions were weekly prepared by dissolution in 100% cell-culture grade, sterile-filtered DMSO (Sigma-Aldrich, Barcelona, Spain; Cat No D2650) and stored at −20 °C between uses. ICI 182,780 (Cat. No. 1047) was obtained from Tocris Cookson Ltd. (Avonmouth, UK). T0070907 (Cat. No. HY-13202) and rosiglitazone (Cat. No. HY-117287) were obtained from MedChem Express (Monmouth Junction, NJ, USA). Due to its short half-life, T0070907 was redosed every 8–10 h. Recombinant human IL-1β and recombinant rat IFNγ were obtained from R&D Systems (Abingdon, UK); (R&D Systems); human IFNγ was obtained from PeproTech (Rocky Hill, NJ). The concentrations of cytokines used herein were selected based on previous dose-response experiments performed in human and rat β-cells [88,89,90].

### 4.2. Culture of INS-1E and EndoC-βH1 Cells

Rat insulin-producing INS-1E cells (RRID: CVCL_0351, kindly provided by Dr. C. Wollheim, Department of Cell Physiology and Metabolism, University of Geneva, Geneva, Switzerland) were cultured in RPMI 1640 GlutaMAX-I, 10 mM HEPES, 1 mM sodium pyruvate, 5% fetal bovine serum, 50 μM 2-mercaptoethanol, 50 units/mL penicillin and 50 mg/mL streptomycin [91]. Human insulin-producing EndoC-βH1 cells (RRID: CVCL_L909, Univercell-Biosolutions, France), which grow attached to Matrigel/fibronectin-coated plates, were cultured in DMEM containing 5.6 mM glucose, 10 mM nicotinamide, 6.7 ng/mL selenite, 5.5 μg/mL transferrin, 50 μM 2-mercaptoethanol, 2% BSA fatty acid free, 100 U/mL penicillin and 100 μg/mL streptomycin [13]. For treatments in EndoC-βH1 cells, 2% fetal bovine serum was added to the culture medium. Both cell lines were kept at 37 °C in a humidified atmosphere of 95% O_2_ and 5% CO_2_.

### 4.3. Isolation and Dispersion of Mouse Islets

Adult male mice (12–14-weeks-old), which were kept under standard housing conditions (12 h light/dark cycle, food ad libitum), were sacrificed and pancreatic islets were isolated as described [92]. Islets were dispersed into single cells and cultured in polylysine-coated plates as described before [79,93]. Dispersed cells were kept at 37 °C in a humidified atmosphere of 95% O_2_ and 5% CO_2_ and used within 48 h of culture. Experimental procedures were performed according to the Spanish Royal Decree 1201/2005 and the European Community Council directive 2010/63/EU. The ethical committee of Miguel Hernandez University reviewed and approved the methods used herein (approvals ID: UMH-IB-AN-01-14 and UMH-IB-AN-02-14).

### 4.4. Assessment of Cell Viability by MTT Assay

The MTT compound (3-(4,5-dimethylthiazol-2-yl)-2,5-diphenyltetrazolium bromide) (Sigma-Aldrich, Barcelona, Spain) was dissolved in RPMI 1640 without phenol red. MTT was added to each well (0.5 mg/mL) and incubated for 3 h at 37 °C. After incubation, the supernatant was removed by aspiration and 100 µL DMSO was added to dissolve formazan crystals. The absorbance was measured at 595 nm using an iMark™ Microplate Absorbance Reader (Bio-Rad, Hercules, CA, USA) and the percentage of cell viability was calculated.

### 4.5. Assessment of Cell Viability by DNA-Binding Dyes

Percentage of living, apoptotic and necrotic cells was determined after staining with DNA-binding dyes Hoechst 33342 (HO) and propidium iodide (PI) as described [91,94]. Two different observers, one of them being unaware of sample identity to avoid bias, counted a minimum of 500 cells per experimental condition (agreement between results from both observers was >90%). Results are expressed as percentage of apoptosis.

### 4.6. Caspase 3/7 Activity Assay

Caspase 3/7 activity was determined using the Caspase-Glo^®^ 3/7 assay (Promega, Madison, WI, USA) according to the manufacturer’s instructions. Briefly, upon 48 h treatment in 100 µL culture medium, cells were incubated with 100 µL Caspase-Glo^®^ 3/7 reagent at room temperature for 1 h before recording luminescence with a POLASTAR plate reader (BMG Labtech, Germany).

### 4.7. DCF Assay

ROS production was measured using the fluorescent probe 2′,7′-dichlorofluorescein diacetate (DCF; Sigma-Aldrich, Barcelona, Spain). Cells were seeded in 96-well black plates and, upon treatment, were loaded with 10 μM DCF for 30 min at 37 °C. DCF fluorescence was quantified in a POLASTAR plate reader (BMG Labtech, Germany). Data are expressed as DCF fluorescence corrected by total protein. Menadione (15 μM for 90 min) was used as a positive control.

### 4.8. Glucose-Stimulated Insulin Secretion

Cells were preincubated in Krebs-Ringer buffer (115 mM NaCl, 5 mM KCl, 1 mM MgCl_2_, 1 mM CaCl_2_, 24 mM NaHCO_3_, 10 mM HEPES pH 7.4 and 0.1% BSA) for 1 h before glucose stimulation. At the end of this incubation, cells were sequentially stimulated with low (0 mM) and then high glucose (20 mM) for 40 min (each stimulation). After each stimulatory period, the incubation medium was collected, placed onto ice and centrifuged at 700× *g*, 5 min at 4 °C. The supernatant was transferred into a fresh tube and stored at −20 °C until insulin measurements. For insulin content, cells were lysed in 100 µL of cell lysis solution (137 mM NaCl, 1% Glycerol, 0.1% Triton X100, 2 mM EGTA, 20 mM Tris pH 8.0 and protease inhibitor cocktail). Cell lysates were centrifuged at 700× *g*, 5 min at 4 °C. The supernatant was transferred into a fresh tube and stored at −20 °C until insulin measurements. Insulin release and content were measured using a human insulin ELISA kit (Mercodia, Uppsala, Sweden).

The amount of secreted insulin secretion as % of total insulin was calculated as previously described [54]. Data are normalized to insulin secretion at high glucose in vehicle-treated cells.

### 4.9. RNA Extraction and Real-Time PCR

Total RNA was isolated using the RNeasy Micro Kit (Qiagen) and poly(A)^+^ mRNA extraction was performed using Dynabeads mRNA DIRECT kit (Invitrogen) in accordance with the manufacturer’s instructions. cDNA synthesis was performed using the High-Capacity cDNA Reverse Transcription Kit (Applied Biosystems). Quantitative PCR was carried out using the CFX96 Real Time System (Bio-Rad) as described [80]. Gapdh and β-actin were used as housekeeping genes for rat and human samples, respectively. The CFX Manager Version 1.6 (Bio-Rad, Hercules, CA, USA) was used to analyze the values, which were expressed as relative expression. The primers used herein are listed in Supplementary Table S1.

### 4.10. Data Analysis

The GraphPad Prism 7.0 software (GraphPad Software, La Jolla, CA, USA) was used for statistical analyses. Shapiro–Wilk normality test was used to determine the normal distribution. Data are presented as the mean ± SEM. Statistical analyses were performed using Student’s *t*-test, one-way ANOVA or two-way ANOVA as stated in the figure legends. One- and two-way ANOVA were followed by Dunnett’s test as post hoc analysis. *p* values ≤ 0.05 were considered statistically significant.

## Data Availability

Not applicable.

## Supplementary Materials

The following supporting information can be downloaded at: https://www.mdpi.com/article/10.3390/ijms23095040/s1.
