# Supplementary material for: In Vitro Assays to Identify Metabolism-Disrupting Chemicals with Diabetogenic Activity in a Human Pancreatic β-Cell Model
Source: Int J Mol Sci. 2022 May 1;23(9):5040. doi: 10.3390/ijms23095040 (PMC9102687; doi:10.3390/ijms23095040)
Supplement: Supplementary file 1 [file ijms-23-05040-s001.zip › ijms-1674178-supplementary.pdf]

International Journal of *Molecular Sciences*

MDPI

Article

In Vitro Assays to Identify Metabolism-Disrupting Chemicals with Diabetogenic Activity in a Human Pancreatic β-Cell Model

Supplementary Material

Table S1. List of primers used in this study.

Gene	Accession number	Primer direction	Primer sequence (5'-3')	Amplicon size (bp)	Annealing temperature (°C)
**Human β-actin (*ACTB*)**	NM_001101.5	Forward	CTGTACGCCAACACAGTGCT	127	63.3
		Reverse	GCTCAGGAGGAGCAATGATC		
**Human *INS***	NM_000207.3	Forward	GCTTCTTCTACACACCCAAGAC	138	63.7
		Reverse	CCACAATGCCACGCTTCT		
**Human *PDX1***	NM_000209.4	Forward	AAAGCTCACGCGTGGAAA	145	63.2
		Reverse	GCCGTGAGATGTACTTGTTGA		
**Human *MAFA***	NM_201589.3	Forward	TACAGGACGTGGACACCA	142	63.0
		Reverse	GTTCTCCGCTCAACCTCAG		
**Human *GLUT2* (*SLC2A2*)**	NM_000340.2	Forward	TTCACTGCTGTCTCTGTATTCC	100	62.9
		Reverse	CTGACATGAAGATGGCACAAAC		
**Human *GCK***	NM_000162.5	Forward	GTCACCTGCAGCCTAATTACT	90	62.9
		Reverse	GCTTAGTGTCCTTCAGACAGATT		
**Rat *Gapdh***	NM_017008.4	Forward	AGTTCAACGGCACAGTCAAG	118	63.7
		Reverse	TACTCAGCACCAGCATCACC		
**Rat *Ins1***	NM_019129.3	Forward	ACCTTTGTGGTCCTCACCTG	118	64.9
		Reverse	AGCTCCAGTTGTGGCACTTG		
**Rat *Ins2***	NM_019130.2	Forward	TGTGGTTCTCACTTGGTGGA	111	64.3
		Reverse	CTCCAGTTGTGCCACTTGTG		
**Rat *Pdx1***	NM_022852.3	Forward	GGTATAGCCAGCGAGATGCT	153	63.9
		Reverse	TCAGTTGGGAGCCTGATTCT		
**Rat *Mafa***	NM_001399773.1	Forward	AAGGAGGAGGTCATCCGACT	127	65.2
		Reverse	TCTGGAGCTGGCACTTCTCG		
**Rat *Glut2* (*Slc2a2*)**	NM_012879.2	Forward	TCAGCCAGCCTGTGTATGCA	79	67.0
		Reverse	TCCACAAGCAGCACAGAGACA		
**Rat *Gck***	NM_001270849.1	Forward	AGACTGACTATCCGGCTACAT	100	63.1
		Reverse	CCCAGAACTGTAAGCCACTC		

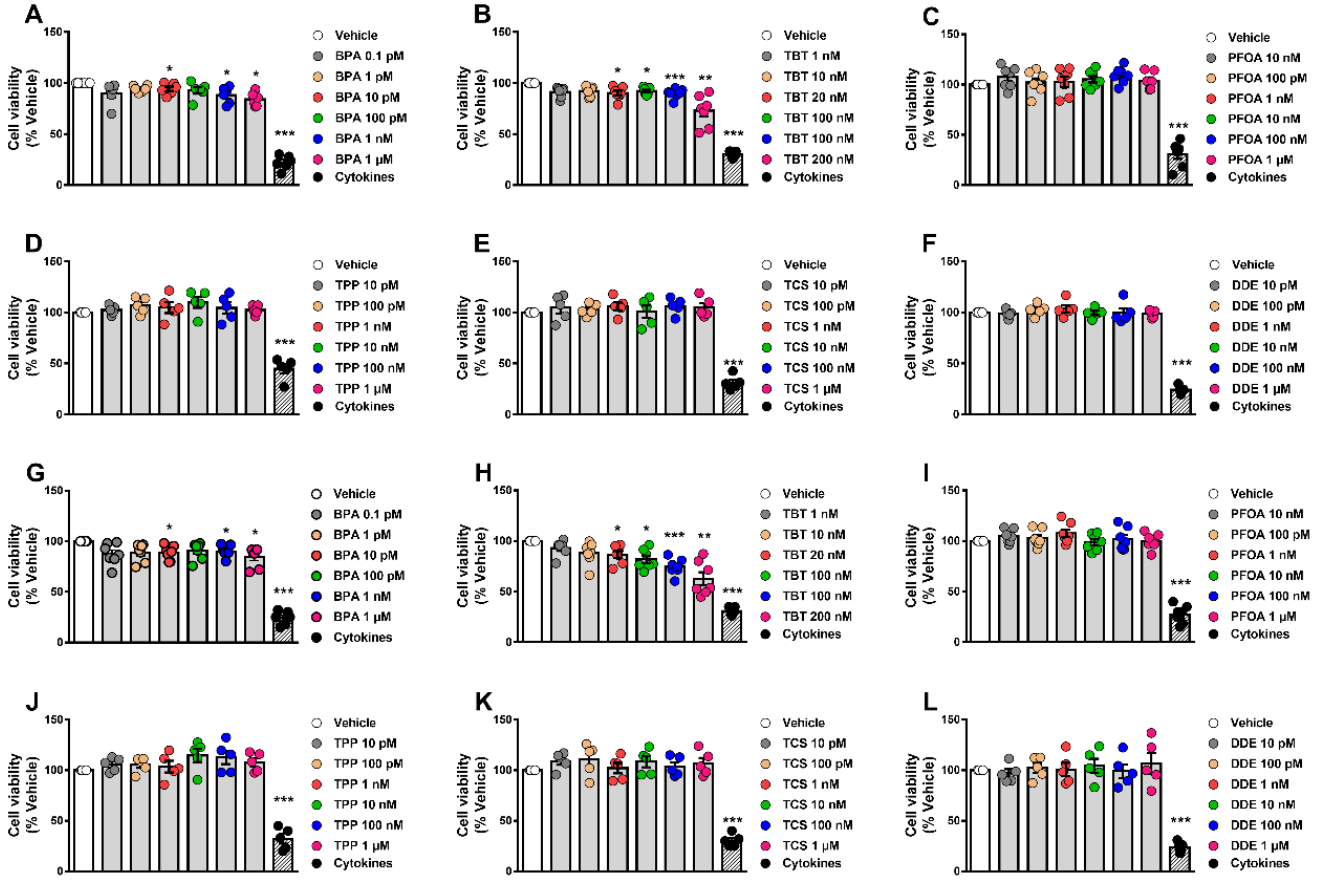

Figure S1. β-cell viability upon MDC exposure. INS-1E cells were treated with vehicle (DMSO) or different doses of BPA (A,G), TBT (B,H), PFOA (C,I), TPP (D,J), TCS (E,K), or DDE (F,L) for 48 h (A-F) or 72 h (G-L). A cocktail of the cytokines IL-1β + IFNγ (10 and 100 U/ml, respectively) was used as a positive control. Cell viability was evaluated by MTT assay. Results are expressed as % vehicle-treated cells. Data are shown as means ± SEM (*n* = 5-7 independent experiments, where each dot represents an independent experiment). *p≤0.05, **p≤0.01 and ***p≤0.001 vs Vehicle. MDCs vs Vehicle by one-way ANOVA; Cytokines vs Vehicle by two-tailed Student's t test.

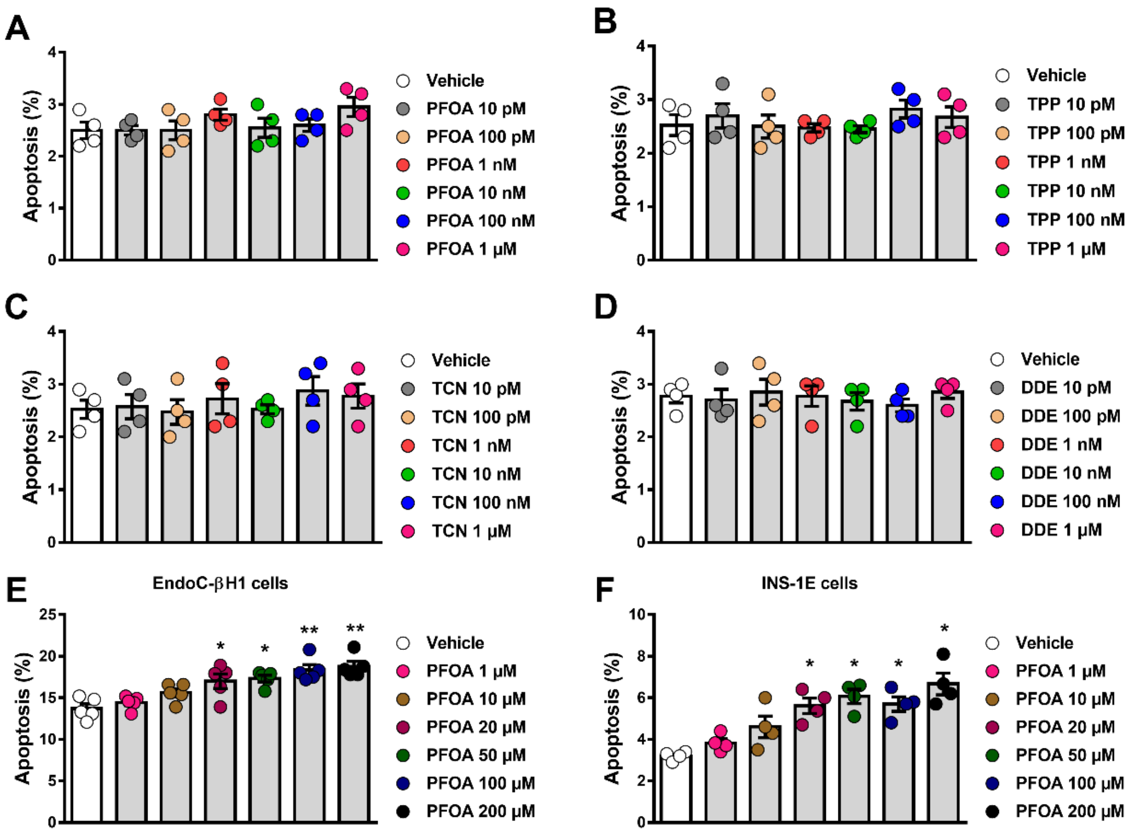

Figure S2. β-cell apoptosis upon MDC exposure. INS-1E (A-D, F) and EndoC-βH1 (E) cells were treated with vehicle (DMSO) or different doses of PFOA (A), TPP (B), TCS (C), DDE (D) for 24 h. Apoptosis was evaluated using HO and PI staining. Data are shown as means ± SEM (*n* = 4-5 independent experiments, where each dot represents an independent experiment). *p≤0.05 and **p≤0.01 vs Vehicle. MDCs vs Vehicle by one-way ANOVA.

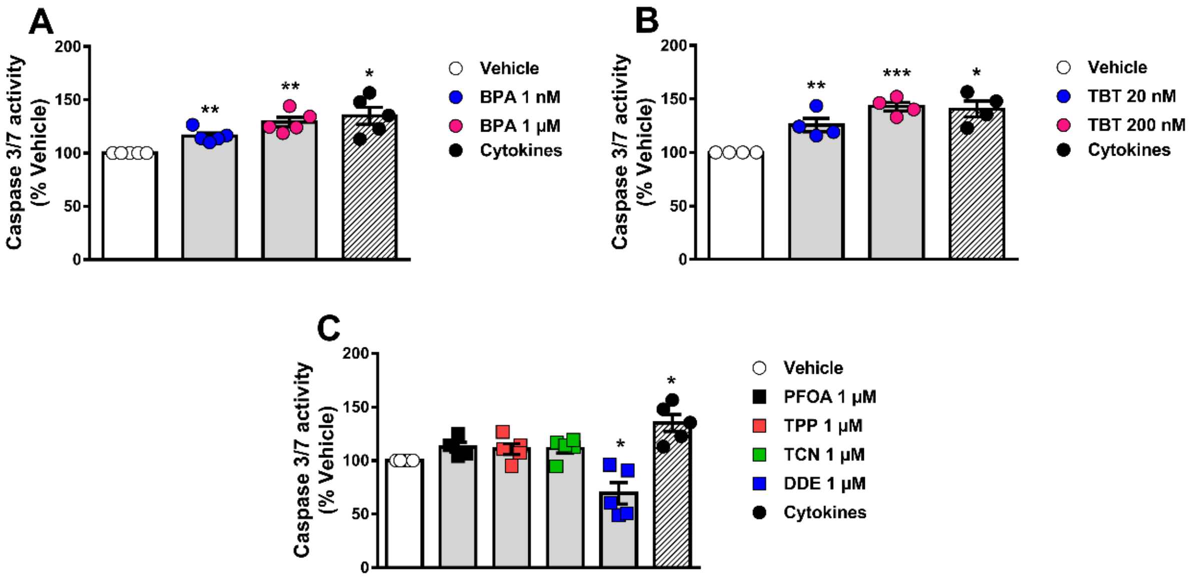

Figure S3. Caspase 3/7 activity upon MDC exposure. EndoC-βH1 cells were treated with vehicle (DMSO) or different doses of BPA (A), TBT (B), PFOA, TPP, TCS, or DDE (C) for 48 h. A cocktail of the cytokines IL-1β + IFNγ (50 and 1000 U/ml, respectively) was used as a positive control. Caspase 3/7 activity was measured by a luminescent assay. Results are expressed as % vehicle-treated cells. Data are shown as means ± SEM (*n* = 4-5 independent experiments, where each dot represents an independent experiment). *p≤0.05, **p≤0.01 and ***p≤0.001 vs Vehicle. MDCs vs Vehicle by one-way ANOVA; Cytokines vs Vehicle by two-tailed Student's t test.

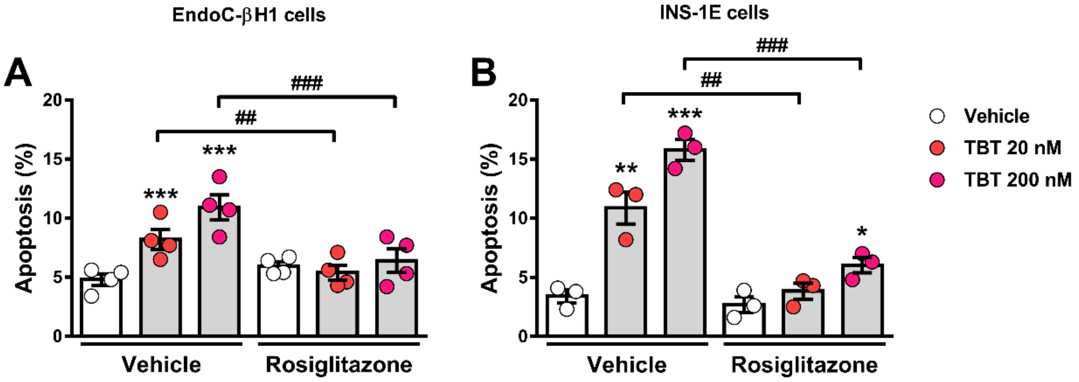

Figure S4. PPARγ is involved in TBT-induced β-cell apoptosis. EndoC-βH1 (A) and INS-1E (B) cells were treated with vehicle (DMSO) or TBT (20 nM or 200 nM) in the absence or presence of 10 μM rosiglitazone for 24 h. Apoptosis was evaluated using HO and PI staining. Data are shown as means ± SEM (*n* = 3-4 independent experiments, where each dot represents an independent experiment). *p≤0.05, **p≤0.01 and ***p≤0.001 vs its respective Vehicle; ##p≤0.01, and ###p≤0.001 as indicated by bars. Two-way ANOVA.

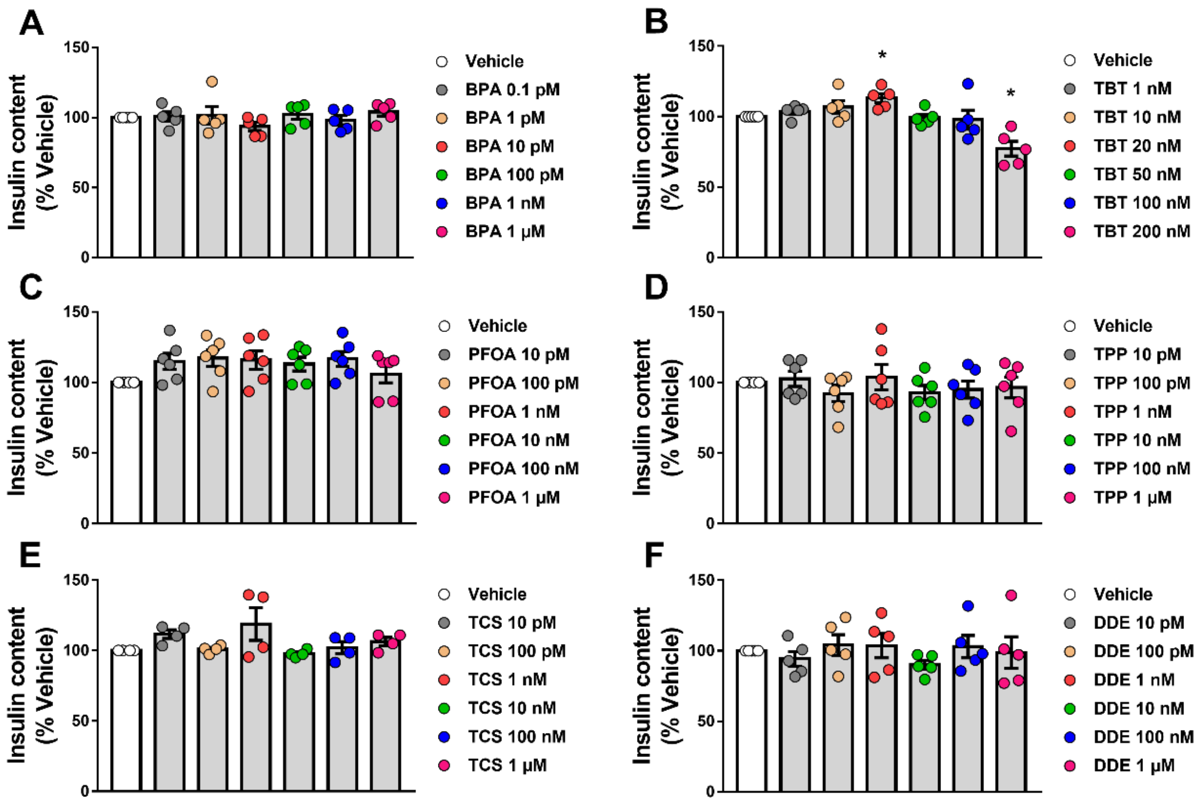

Figure S5. Insulin content upon MDC exposure. EndoC-βH1 cells were treated with vehicle (DMSO) or different doses of BPA (A), TBT (B), PFOA (C), TPP (D), TCS (E), or DDE (F) for 48 h. Insulin content was measured by ELISA. Results are expressed as % vehicle-treated cells. Data are shown as means ± SEM (n = 4-6 independent experiments, where each dot represents an independent experiment). *p≤0.05 vs its respective Vehicle. One-way ANOVA.

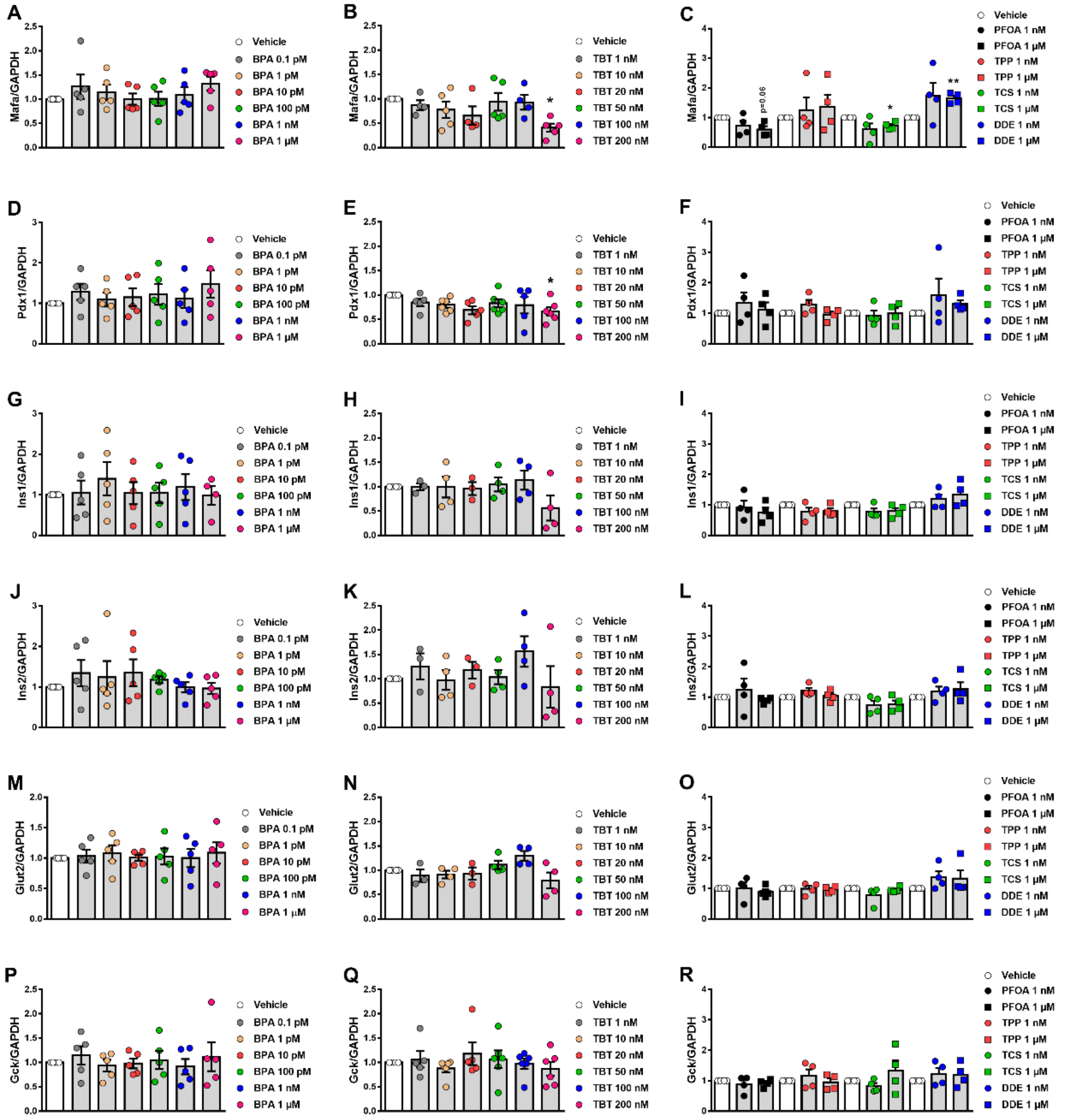

Figure S6. Gene expression upon MDC exposure. mRNA expression of *Mafa* (A-C), *Pdx1* (D-F), *Ins1* (G-I), *Ins2* (J-L), *Glut2* (M-O), and *Gck* (P-R) was measured in INS-1E cells treated with vehicle (DMSO) or different doses of BPA (A, D, G, J, M and P), TBT (B, E, H, K, N and Q), PFOA, TPP, TCS, or DDE (C, F, I, L, O and R) for 24 h. mRNA expression was measured by qRT-PCR and normalized to the housekeeping gene *Gapdh*, and then by vehicle-treated cells (considered as 1). Data are shown as means ± SEM (*n* = 3-6 independent experiments, where each dot represents an independent experiment). *p≤0.05 and **p≤0.01 vs its respective Vehicle. One-way ANOVA.
